# High glucose concentration up-regulates the expression of matrix metalloproteinase-9 and -13 in tendon cells

**DOI:** 10.1186/1471-2474-14-255

**Published:** 2013-08-27

**Authors:** Wen-Chung Tsai, Fang-Chen Liang, Ju-Wen Cheng, Li-Ping Lin, Shih-Chieh Chang, Hsiang-Hung Chen, Jong-Hwei S Pang

**Affiliations:** 1 Department of Physical Medicine and Rehabilitation, Chang Gung Memorial Hospital at Linkou, Taoyuan, Taiwan; 2 College of Medicine, Chang Gung University, Taoyuan, Taiwan; 3 School of Medicine, Chung Shan Medical University, Taichung, Taiwan; 4 Graduate Institute of Clinical Medical Sciences, Chang Gung University, Taoyuan, Taiwan

**Keywords:** Glucose, Matrix metalloproteinase, Tendon

## Abstract

**Background:**

Diabetes mellitus is associated with tendinopathy or tendon injuries. However, the mechanism underlying diabetic tendinopathy is unclear. The purpose of this study was to examine the effects of high glucose concentrations on the activity and expression of matrix metalloproteinases, type I collagen, and type III collagen in tendon cells.

**Methods:**

Tendon cells from rat Achilles tendons were treated with 6 mM, 12 mM, and 25 mM glucose, and then cell proliferation was evaluated by the 3-[4,5-Dimethylthiazol-2-yl]-2,5-diphenyltetrazolium bromide (MTT) assay. Messenger RNA (mRNA) expression of MMP-2, MMP-8, MMP-9, and MMP-13 and type I and type III collagen was assessed by quantitative real-time polymerase chain reaction (PCR). The enzymatic activity of MMP-2 and MMP-9 was measured by gelatin zymography.

**Results:**

The MTT assay results showed that the glucose concentration did not affect tendon cell proliferation. The results of the real-time PCR assay revealed that the mRNA expression of MMP-9 and MMP-13 was up-regulated by treatment with 25 mM glucose, whereas the mRNA expression of type I and III collagen was not affected. Gelatin zymography showed that 25 mM glucose increased the enzymatic activity of MMP-9.

**Conclusions:**

High glucose concentration up-regulates the expression of MMP-9 and MMP-13 in tendon cells, which may account for the molecular mechanisms underlying diabetic tendinopathy.

## Background

Diabetes mellitus is associated with many musculoskeletal disorders such as tenosynovitis, adhesive capsulitis, calcific tendinopathy, and limited joint mobility [1-4]. Musculoskeletal disorders of the hand and shoulder are reportedly more common in diabetic patients than in non-diabetic patients [5]. However, few studies have investigated the mechanisms underlying the pathogenesis of diabetic tendinopathy.

The tendon consists of tendon cells (fibroblasts) and collagens. Tendon cells provide collagen, protein mediators for tendon repair, and matrix proteoglycans [6]. Approximately 90% of the collagen in normal tendons is type I, and less than 10% is type III [7]. The biomechanical properties of a tendon are primarily a feature of the extracellular matrix (ECM), which is in a constant state of dynamic equilibrium between synthesis and degradation [8].

Matrix metalloproteinases (MMPs) are a family of ECM-degrading enzymes, which are inhibited by a family of proteins called the tissue inhibitors of MMPs (TIMPs) [9]. MMPs are important regulators of ECM network remodeling, and MMP levels are altered during tendon healing [9-11]. MMP-8 and MMP-13 are the collagenases that cleave type I collagen molecules in the ECM [12,13]. MMP-2 and MMP-9 also have collagenolytic activity [13,14]. Previous findings suggest that MMP-9 and MMP-13 only participate in collagen degradation, whereas MMP-2, MMP-3, and MMP-14 participate in both collagen degradation and remodeling [11,15]. Therefore, the activities of MMPs may play an important role in tendon healing. The potential combination of increased local matrix degradation due to enhanced MMP expression and decreased ECM production by tendon cells in high glucose concentrations might predispose patients with diabetes to tendinopathy or tendon rupture.

Many studies have investigated the effects of high glucose on MMP expression in different cell lines. High glucose concentrations increased the expression of MMP-9 in primary cultured neurons as well as the activities of MMP-2 and MMP-9 in endothelial cells [16,17]. High glucose concentrations also induced MMP-9 promoter activity, mRNA expression, protein expression, and gelatinase activity in bovine aortic endothelial cells [18]. High glucose concentrations also enhanced the expression and activity of MMP-2 in smooth muscle cells and adventitial fibroblasts [19,20]. In addition, diabetic rats exhibited a longer duration of increased MMP-1 than did non-diabetic rats [21]. However, few studies have investigated the effects of high glucose concentration on the expression of MMPs in tendon cells.

We hypothesized that diabetic tendinopathy is related to the up-regulation of MMPs in tendon cells due to the high glucose environment. The purpose of this study was to investigate the effects of high glucose on the activity and expression of MMPs and the expression of type I and III collagen in tendon cells.

## Methods

### Primary culture of rat Achilles tendon cells

All experimental procedures were conducted in accordance with the Guide for the Care and Use of Laboratory Animals and were approved by the Animal Care and Use Committee of Chang Gung Memorial Hospital. The Achilles tendons from 16 Sprague–Dawley rats (weighing 200–250 g) around 7–8 weeks old were excised. The excised tendons were soaked in povidone-iodine for 3 minutes and washed twice with phosphate-buffered saline (PBS). Each tendon was then cut into small pieces approximately 1.5–2.0 mm^3^ (6 pieces in total). Each piece was individually placed in a well of 6-well culture plates.

After 5 minutes of air-drying for better adherence, 0.5 mL of Dulbecco’s modified Eagle’s medium (DMEM; HyClone, Logan, UT, USA), with 10% fetal bovine serum (Cansera, Rexdale, Ontario, Canada), 100 U/mL penicillin, and 100 μg/mL streptomycin were added to each well. The explants were then incubated at 37°C in a humidified atmosphere of 5% CO_2_/95% air. After migrating from the explants, the cells started to grow rapidly and the confluent culture was then subcultured at a 1:3 dilution after trypsin digestion. Tendons cells with proper growth rates and normal fibroblast-shapes were used in the following experiments.

### MTT (3-[4,5-Dimethylthiazol-2-yl]-2,5-diphenyltetrazolium bromide; Thiazolyl blue) assay

The MTT assay was used to measure cell survival and proliferation. Tendon cells were first seeded in 24-well flat-bottomed culture plates. Then the cells were treated with medium containing different concentrations of glucose (6 mM, 12 mM, and 25 mM). Twenty-four hours later, the cells were observed under a microscope and then the MTT assay was performed. DMEM, containing 50 μg/mL MTT, was added to each well and the plate was incubated at 37°C for 1 hour. Then, the MTT solution was removed and 1 mL of dimethyl sulfoxide (DMSO) was added to each well. After the crystals were dissolved by mixing with micropipette, the colorless DMSO turned purple. Aliquots were transferred to a 96-well plate and then read immediately at 570 nm in a scanning multi-well spectrophotometer. These experiments were performed in triplicate.

### Quantitative real-time polymerase chain reaction (PCR) analysis

Total RNA was extracted from tendon cells using solution D (1 mL of solution D/10^7^ cells). Subsequently, total RNA was extracted with phenol and chloroform/isoamyl alcohol (49:1) to remove proteins and genomic DNA. Complementary (c) DNA was synthesized using 1 mg of total RNA in a 20 mL RT reaction mix containing 0.5 mg of random primers, 0.8 mM dNTPs, 0.1 M DTT, and 1 L of first strand buffer. Quantitative real-time PCR was performed using SYBR Green and MxPro-Mx3000P QPCR machine (Stratagene, NeoMarkers, Fremont, CA, USA). Aliquots (20 ng) of cDNA were used for each quantitative real-time PCR, and each reaction was performed in triplicate. The relative gene expression between experimental groups was calculated using MxPro software (Stratagene) and glyceraldehyde-3-phosphate dehydrogenase (GAPDH) was used as an internal control. All real-time PCRs were performed in triplicate, and changes in gene expression were reported as multiples of increases relative to the untreated controls. The oligonucleotide sequences of the specific primers used in this study are shown in Table 1.

**Table 1:** Oligonucleotide sequences for the specific primers used in real time PCR

GADPH	
	Sense: AGTCTACTGGCGTCTTCA
	Antisense: TTGTCATATTTCTCGTGGT
MMP-2	
	Sense: GGAAGCATCAAATCGGACTG
	Antisense: GGGCGGGAGAAAGTAGCA
MMP-8	
	Sense: GCCCGACTCTGGTGATTT
	Antisense: TGATGTCTGCTTCTCCCT
MMP-9	
	Sense: CCCACTTACTTTGGAAACG
	Antisense: GAAGATGAATGGAAATACGC
MMP-13	
	Sense: CCACCTTCTTCTTGTTGA
	Antisense: GCATTTCTCGGAGTCTAT
Type I collagen	
	Sense*:* TGGAGACAGGTCAGACCTG
	Antisense: TATTCGATGACTGTCTTGCC
Type III collagen	
	Sense*:* TAAAGGGTGAACGGGGCAGT
	Antisense: ACGTTCCCCATTATGGCCAC

### Gelatin zymography

The tendon cells were treated with medium containing different glucose concentrations (6 mM, 12 mM, and 25 mM) for 24 hours. MMP-2 and MMP-9 in the conditioned medium were detected by gelatin zymography, which was performed under non-reducing conditions using a 7.5% SDS polyacrylamide gel containing 2 mg/mL gelatin (Mini-PROTEAN II system; Bio-Rad Laboratories Ltd, Hempstead, UK). The gels were washed in 2.5% Triton X-100 to remove the SDS and allow renaturation of the MMPs, and then transferred to a buffer containing 50 mM Tris (pH 7.5), 5 mM CaCl_2_, and 1 mM ZnCl_2_ and incubated at 37°C for 18 hours. After staining with Coomassie brilliant blue R250 (Bio-Rad Laboratories), white bands indicative of gelatin degradation by pro-MMPs and active MMPs were detected.

### Statistical analysis

All nonparametric data obtained were expressed as the means ± standard error of the mean (SEM). The values among the 3 groups were compared using the Kruskal–Wallis test. Differences between groups were measured by the Mann–Whitney test. P-values less than 0.05 were considered statistically significant.

## Results

**Figure 1:**
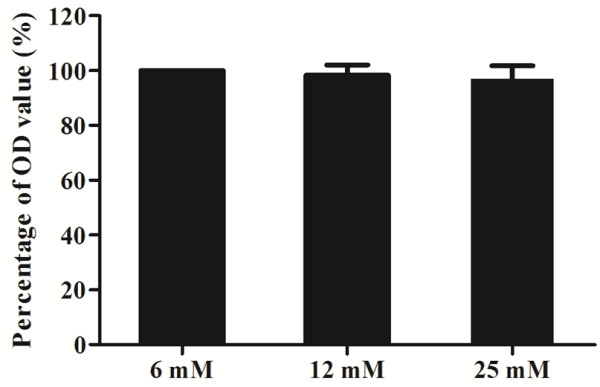
**MTT assay for the effect of glucose on tendon cells.** A high glucose concentration did not affect the proliferation of tendon cells (*p* > 0.05 by Kruskal-Wallis test, n = 3; X-axis, glucose concentration; Y-axis, percentage of optic density (OD) value).

The MTT assay for tendon cell viability showed that tendon cells treated with 6 mM, 12 mM, and 25 mM glucose had similar optic density (OD) values. The OD values were 100% in the 6 mM glucose group, 99.0% ± 3.6% in the 12 mM glucose group, and 97.0% ± 4.8% in the 25 mM glucose group (*p* > 0.05; Figure 1).

### mRNA expression of MMP-2, MMP-8, MMP-9, and MMP-13

**Figure 2:**
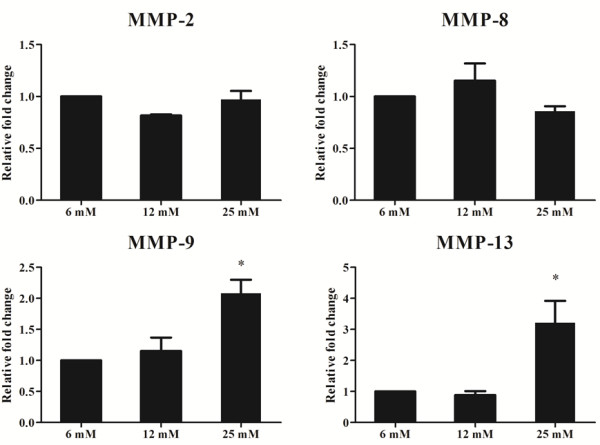
Real-time PCR assay to assess the effect of a high glucose concentration on the mRNA expression of MMP-2, MMP 8, MMP-9, and MMP-13 (*p **< 0.05 by Kruskal-Wallis test, n = 3; X-axis, glucose concentration; Y-axis, relative fold change).**

The results of real-time PCR showed that the mRNA expression of MMP-9 and MMP-13 was up-regulated by 25 mM glucose. However, the mRNA expression of MMP-2 and MMP-8 was not affected by glucose concentration (Figure 2).

The relative fold changes in the levels of MMP-2 mRNA in cells treated with 6 mM, 12 mM, and 25 mM glucose medium were 1, 0.82 ± 0.01, and 0.97 ± 0.08, respectively (*p* > 0.05). The relative fold changes in the levels of MMP-8 mRNA in cells treated with 6 mM, 12 mM, and 25 mM glucose medium were 1, 1.15 ± 0.17, and 0.86 ± 0.05, respectively (*p* > 0.05). In contrast, the relative fold changes in the levels of MMP-9 mRNA in cells treated with 6 mM, 12 mM, and 25 mM glucose medium were 1, 1.16 ± 0.22, and 2.08 ± 0.22, respectively, and this difference was statistically significant (*p* = 0.008). The relative fold changes in the levels of MMP-13 mRNA in cells treated with 6 mM, 12 mM, and 25 mM glucose medium were 1, 0.88 ± 0.13, and 3.22 ± 0.70, respectively (*p* = 0.024). The difference between the 6 mM group and the 25 mM group was statistically significant (*p* = 0.03).

### mRNA expression of type I & III collagen

**Figure 3:**
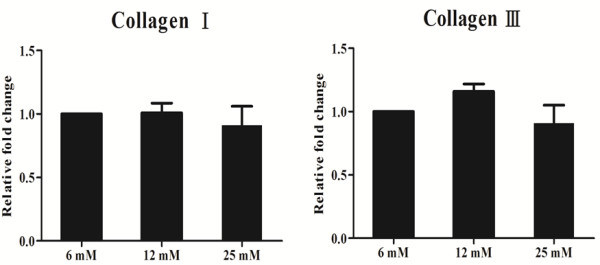
**Real-time PCR assay to assess the effect of a high glucose concentration on the mRNA expression of type I and III collagen.** A high glucose concentration had no effect on the mRNA expression of type I and III collagen (*p* > 0.05 by Kruskal-Wallis test, n = 3; X-axis, glucose concentration; Y-axis, fold change relative to expression in 6 mM glucose).

The real-time PCR results revealed that the mRNA expression of type I and III collagen was not affected by glucose concentration (*p* > 0.05; Figure 3). The relative mRNA levels of type I collagen in cells treated with 6 mM, 12 mM, and 25 mM glucose were 1, 1.01 ± 0.08, and 0.91 ± 0.15, respectively. The relative mRNA levels of type III collagen in cells treated with 6 mM, 12 mM, and 25 mM glucose were 1, 1.16 ± 0.06, and 0.91 ± 0.14, respectively. The expression of type I and III collagen decreased nearly 10% in tendon cells treated with 25 mM glucose compared to their expression in cells treated with 12 mM glucose.

### Enzymatic activities of MMP-2 and MMP-9

**Figure 4:**
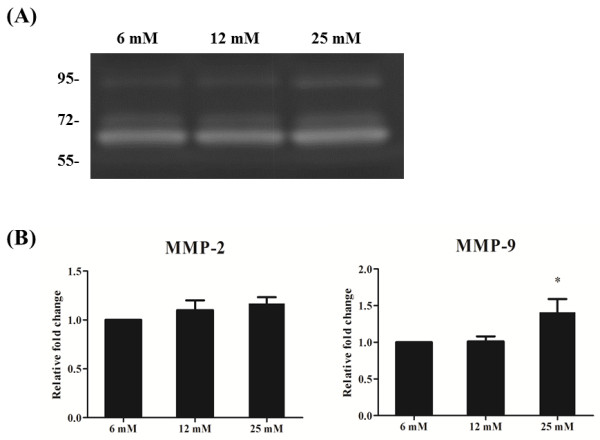
**Gelatin zymography for the effect of glucose on tendon cells. (A)** Gelatin zymography showed that MMP-2 and MMP-9 had enzymatic activities at a molecular size of 72 kDa and 95 kDa, respectively. **(B)** Densitometric analysis of the MMP-2 and MMP-9 zymogram revealed that the enzymatic activity of MMP-9 in tendon cells increased after glucose treatment (*p < 0.05, n = 3).

Gelatin zymography revealed that the enzymatic activity of MMP-9 increased in higher glucose concentrations (Figure 4A). Furthermore, densitometric analysis showed that the relative MMP-9 enzymatic activity in tendon cells treated with 12 mM and 25 mM glucose was 1.02 ± 0.07 and 1.41 ± 0.18, respectively, relative to the activity in tendon cells treated with 6 mM glucose. The difference between the MMP-9 activity in 6 mM glucose and 25 mM glucose was statistically significant (*p* < 0.05; Figure 4B). The relative MMP-2 enzymatic activity in tendon cells treated with 12 mM and 25 mM was 1.10 ± 0.10 and 1.20 ± 0.10, respectively, relative to the activity in tendon cells treated with 6 mM glucose. This trend of increasing MMP-2 enzymatic activity in higher concentrations of glucose indicated that MMP-2 activity was enhanced by glucose treatment in a dose-dependent manner. However, the difference was not statistically significant (*p* > 0.05; Figure 4B).

## Discussion

To the best of our knowledge, this study is the first to document that a high glucose concentration could up-regulate the mRNA expression of MMP-9 and MMP-13 as well as the enzymatic activity of MMP-9 in tendon cells. These novel findings may explain, in part, the molecular mechanisms underlying diabetes associated tendinopathy. It was previously reported that sustained hyperglycemia impairs tendon-bone healing after rotator cuff repair in a rodent model [22]. In addition to the hyperglycemic condition, advanced glycation end products (AGEs), resulting from the exposure of proteins to high levels of sugar, prematurely accumulate in the long-lived proteins of connective tissues, including tendons [23]. These may also be involved in diabetes mellitus-associated tendinopathy. AGE-modified proteins also affect ECM production at the cellular level. By signaling through cell surface receptors, they can alter the ECM profile synthesized by resident connective tissue cells [24,25]. It has been postulated that diabetes negatively impacts the tendon ECM profile not only due to AGE formation but also as a result of increased MMP-9 and MMP-13 expression in tendon cells. These aforementioned mechanisms may weaken the tendon’s mechanical properties, which could lead to tendinopathy or tendon rupture.

Studies investigating the in vitro effects of hyperglycemia on the proliferation of various cell lines have yielded diverse results. High concentrations of glucose inhibit the proliferation of and type I collagen secretion from human periodontal ligament fibroblasts. [26]. However, glucose initially stimulates peritoneal fibroblast proliferation, and then inhibits proliferation [27]. Under hyperglycemic conditions, the migration and proliferation capacities of keratinocytes were reduced [28]. However, to the best of our knowledge, few studies have investigated the proliferation of tendon cells in a hyperglycemic environment. In our experimental setting, we showed that tendon cell proliferation was not affected by glucose concentrations up to 25 mM.

Our experiment was designed to mimic the normal blood sugar environment in non-diabetics and the hyperglycemic environment in diabetics. The blood sugar level in non-diabetics ranges from 70–105 mg/dL, which is approximately equal to 6 mM glucose, the low concentration of glucose used in our experiments. The 2 higher glucose concentrations we used, 12 mM and 25 mM, are approximately 200 mg/dL and 400 mg/dL, respectively. Therefore, tendon cells treated with 12 mM and 25 mM glucose were used to simulate the environment of tendon cells in diabetic patients.

Previous studies showed that the expression of certain MMPs is correlated with tendon injury and tendon rupture [29]. MMP-2 is expressed and activated during healing of an acute supraspinatus tendon tear [30]. Serum levels of MMP-2, MMP-7, and TIMP-2 are elevated in patients with a history of Achilles tendon rupture [31]. In patients with rheumatoid arthritis, invasive tenosynovitis is associated with an increased rate of tendon rupture due to increased MMP-8 activity [32]. Supraphysiologic levels of the proteinase MMP-8 can degrade collagen, which leads to impaired wound healing in rats [33]. The expression of MMP-1 and MMP-9 is significantly higher in torn cuff samples than in healthy tendons [34]. Increased collagenase-3 (MMP-13) mRNA levels and increased levels of the active form of MMP-13 were noted in rotator cuff tendon tears [35]. Our present study showed that a hyperglycemic environment could up-regulate the mRNA expression of MMP-9 and MMP-13 in tendon cells. In addition, zymography provided further evidence that the enzymatic activity of MMP-9 increased in higher glucose concentrations. It is possible that these effects will induce collagen degradation, which could have a negative impact on the integrity of the ECM of a tendon. Therefore, it would inevitably weaken the mechanical properties of the tendon. These findings might partly explain the mechanism underlying the increased incidence of tendinopathy in patients with diabetes.

There are limitations to this study. First, the glucose concentrations used in this study were based on the studies of Thomas et al., which demonstrated that the glucose concentration in subcutaneous tissue closely resembled that in plasma [36]. However, to our knowledge, there has been no report on the glucose concentration in tendon tissue in the normal population or in diabetic patients. We assumed that the glucose concentration in tendons was similar to that in subcutaneous tissues. However, this assumption needs to be verified. Second, we should be cautious when extrapolating the findings from this in vitro study to in vivo condition. Therefore, further animal studies should be done to validate the effects of high glucose concentration on the expression of MMPs and the regulation of type I and III collagen in tendon cells.

## Conclusions

High glucose concentrations could up-regulate the mRNA expression of MMP-9 and MMP-13 as well as the enzymatic activity of MMP-9 in tendon cells. These findings may partly explain the molecular mechanisms underlying diabetes-associated tendinopathy.

## Competing interests

The authors declare that they have no competing interests.

## Authors’ contributions

WCT and FCL carried out the experiments and drafted the manuscript. JWC and LPL performed the statistical analysis and drafted the manuscript. SCC and HHC did literature review and helped to carry out the experiments. JHSP participated in the design and coordination of the study, and helped to draft the manuscript. All authors read and approved the final manuscript.

## Pre-publication history

The pre-publication history for this paper can be accessed here:

http://www.biomedcentral.com/1471-2474/14/255/prepub
